# The Smart Inertial Device Data from Human Activities dataset

**DOI:** 10.1038/s41597-026-06860-w

**Published:** 2026-04-09

**Authors:** Riccardo Pignari, Bendetto Leto, Stefano Quer, Enrico Macii, Gianvito Urgese, Vittorio Fra

**Affiliations:** Politecnico di Torino, Turin, 10129 Italy

**Keywords:** Health care, Engineering

## Abstract

The proliferation of smart devices with inertial measurement units has driven human activity recognition (HAR) research for several years. However, existing datasets like the Smartphone and Smartwatch Activity and Biometrics Dataset (from the Wireless Sensor Data Mining Lab, i.e., the WISDM Lab) may suffer from non-uniform sampling, missing data, and sensor misalignment. To overcome the limitations mentioned above, we present the Smart Inertial Device Data from Human Activities (SIDDHA) dataset, which is a meticulous reconstruction of the previously described dataset. Our rigorous reconstruction employs a two-phase characterization followed by spline interpolation methods for resampling and filtering, yielding a uniformly sampled and realigned data. An additional key innovation of this process is to include spike-encoded inertial data, generated using eleven distinct encoding techniques. We specifically tailor this process for spiking neural networks and neuromorphic computing. Technical validation confirms SIDDHA’s enhanced quality. Experimental results demonstrate an improved HAR and better accuracy on SIDDHA’s raw data with recurrent architectures such as the Legendre memory unit, and the long short-term memory.

## Background & Summary

The widespread diffusion of smart devices, equipped with an increasing number of sensors^1–3^, has led to a substantial growth in data collection, monitoring, and processing in recent years^4^. This trend is evident in various domains, including the automotive^5^, healthcare^6^, smart home^7^, and sports sector^8^. This extensive cross-domain applicability, alongside its seamless integration into daily life, can be attributed to the transformative miniaturization of sensors^9^, which has enabled the wide diffusion of wearable solutions for non-invasive applications. Among the various tasks associated with wearable sensors, the human activity recognition (HAR) stands out due to its rich value of information and its adaptability to diverse use cases. Beyond its utility in monitoring daily routines, the dependable and prompt classification of user activities holds significant importance, particularly in safety-critical scenarios^10,11^.

The pervasive adoption and straightforward deployment of smart devices have unequivocally positioned wearable sensors at the forefront of HAR research over the past decade^12–17^. Recently, significant progress has been made in developing advanced machine learning (ML) models for more accurate classification, leveraging both traditional ML techniques and deep learning (DL) methods^18,19^. To enhance the efficacy of these classification models and broaden the scope of their functionality, substantial amounts of data are required for training. The quality of such data is crucial^20,21^.

From different reviews focusing on the HAR task^22–24^, it is possible to identify various data sources regarding publicly available datasets. Within the array of available datasets, the Smartphone and Smartwatch Activity and Biometrics Dataset^25,26^, originating from the Wireless Sensor Data Mining (WISDM) Lab. We will subsequently reference this dataset as the WISDM dataset for conciseness. It features a collection of inertial measurement unit (IMU) data derived from accelerometers and gyroscopes found within smartphones and smartwatches. The dataset encompasses 18 distinct activities (non-hand oriented, general hand-oriented, and hand-oriented related to eating tasks), recorded across 51 subjects, as succinctly presented in Table 1. However, as highlighted by Heydarian *et al*.^27^, an in-depth analysis of this dataset exposes critical issues, which include a non-uniform sampling of the data, the absence of some classes, and the misalignment of the samples from accelerometer and gyroscope signals.

**Table 1:** Activity class composition for the WISDM dataset.

Number of subjects:		51			
Number of activities:		18			
Non-hand-oriented activities		Hand-oriented activities (general)		Hand-oriented activities (eating)	
Activity	Label	Activity	Label	Activity	Label
Walking	A	Typing	F	Eating Soup	H
Jogging	B	Brushing Teeth	G	Eating Chips	I
Stairs	C	Playing Catch (Tennis)	O	Eating Pasta	J
Sitting	D	Dribbling (Basketball)	P	Drinking from Cup	K
Standing	E	Writing	Q	Eating Sandwich	L
Kicking (Soccer)	M	Clapping	R		
		Folding Clothes	S		

The dataset consists of 51 subjects performing a total of 18 activities. It is divided into different categories, such as non-hand orientated activities, hand orientated activities (general), and hand orientated activities (eating).

**Fig. 1:**
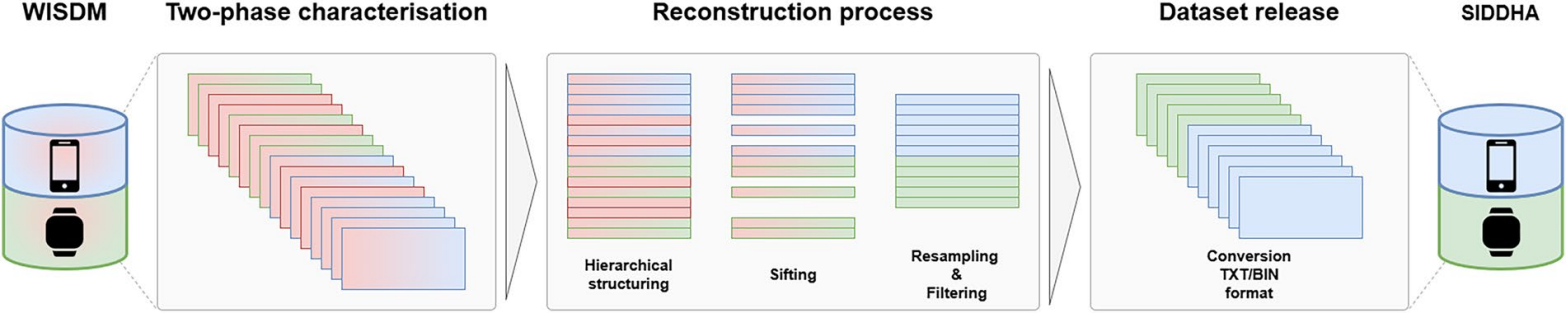
Building blocks of the WISDM sample analysis and the reconstruction process for the SIDDHA production. We employ a two-step characterisation process in the initial phase to identify potential issues affecting individual instances or the entire dataset. In the second phase, we apply the reconstruction process through three steps: hierarchical structuring, which organises the data according to the source device, subject, activity class, and sensor type; sifting, which removes instances with incomplete sensor measurements; resampling and filtering, which reconstruct the signal, providing output instances with uniform sampling at 20 Hz. In the third phase, we convert the reconstructed instances that compose the SIDDHA dataset into binary and text formats.

To meet the need for reliable data that ML and artificial intelligence (AI) models have, we present the Smart Inertial Device Data from Human Activities (SIDDHA) dataset, an improved and updated version of the WISDM dataset^25,26^, produced using a smart reconstruction strategy based on a combination of data analysis, resampling, and filtering. Figure 1 graphically summarizes the characteristics of the reconstruction process. The main target of the dataset is to present high-quality data to ensure the reliability of ML model predictions. For example, in time series data, accurate timestamping is essential to maintaining consistency and alignment of samples across different devices and sources. Moreover, when signals exhibit a uniform frequency and consistent timestamps, their temporal integrity facilitates learning of meaningful patterns^27,28^.

## Methods

We designed a preparatory two-phase characterization process and a reconstruction stage for the WISDM dataset^25,26^ instances. The first phase analyzes the whole data structure using Python. This phase extracts information related to the sampling methods, verifies the available sensor data channels, evaluates the classes’ balancing, and identifies instances with duplicate data. The second phase, using the MATLAB software, involves data interpolation, which is crucial for the subsequent resampling and filtering operations that lead to the instance reconstruction.

### Phase I - Analysis

The analysis step applied to the original dataset consists of exploring and structuring the data based on four structural levels: the source device, the subject who performed the activity, the activity class, and the sensor type. We first perform a characterization based on analyzing the timestamps, the imbalances of the classes, and the sampling frequencies. To explore the timestamps and characterize the samples of the instances, we employ two metrics, i.e., the *temporal sorting* and the *number of timestamps*. We use the former to identify instances where the measurement timestamps occur consecutively without duplication or random sorting. We adopt the latter to represent the number of cases in which we compare the timestamps for the accelerometer and gyroscope to identify any discrepancies between the sensors. The class imbalance within the dataset is quantified through the metrics *missing instance* and *missing channel*. The first allows determining whether a subject is absent from a given class. The second facilitates the identification of cases where an instance is missing one or more channels from the accelerometer or the gyroscope. At the level of individual cases, the investigation concerns the analysis of the sampling frequency for the data provided by the timestamp of the dataset.

The evaluation of these metrics enables a reconstruction strategy based on the different criticalities discovered at the various levels. For example, the *temporal sorting* metric indicates that the subject with id 1629 presents duplicate measurements across all classes from both the smartwatch and the smartphone device. Similarly, the *number of timestamps* metric reveals that over 90.5% of the samples exhibit a discrepancy between the number of measurements recorded by the accelerometer and the gyroscope in the smartphone and 96.7% for the smartwatch. The extent of such misalignment ranges from a minimum of 1 to a maximum of 8936 timestamps. Additionally, the examination of the *missing instance* metric shows that 8 classes are absent from the smartphone device and 6 for the smartwatch. Finally, the *missing channel* metric indicates that 2samples from the smartphone dataset are lacking channels, while 5 in the case of the smartwatch.

### Phase II - Interpolation

In the second phase of the preparatory investigation, we evaluate the efficacy of various interpolation techniques for the signal resampling process. By adopting the resample library provided by MATLAB, we experimented with the following interpolation strategies: linear method, piecewise cubic Hermite interpolating polynomial (PCHIP), and cubic spline. We compare the original and resampled signals to evaluate these approaches and identify the resampling method that produces the least distortion during the process. Unfortunately, a direct comparison, based on the Euclidean distance or the cosine similarity, is not a viable approach due to the different timestamps obtained after sampling the original and the processed signal. Consequently, we quantify the quality of resampling by adopting the dynamic time warping (DTW) and the *signal integral*. The results gathered for the smartphone and the smartwatch samples highlight that the spline technique represents the optimal approach. Using this method and considering the DTW, 72.0% of the reconstructed samples are structurally close to the original ones, and the *signal integral* provides the same outcome with 85.4%.

After the resampling phase, we implement a filtering process to eliminate any frequency components that may potentially give rise to an aliasing problem. We select the Butterworth filter from the filters available in MATLAB’s *Filter Designer* tool to minimize the distortion introduced during the filtering process. We select all parameters coherently with the Nyquist-Shannon sampling theorem^29^ and implement a low-pass filter with a cut-off frequency of 8 Hz, a stop band at 10 Hz, and 100 dB attenuation.

### Reconstruction

After completing the two-step preparatory investigation, we perform the reconstruction process. Similarly to the above-described procedure, we use a twofold approach by relying on both Python and MATLAB to adopt the optimal tools and libraries. As a first step, all the instances gathered from the source files of the WISDM dataset^25,26^ are reorganised, through Python, based on the following hierarchical structure: the device, namely a smartphone or a smartwatch, is the highest level; then, we consider the subject, the activity, and the sensor type (i.e., accelerometer or gyroscope). Following this reorganisation, we implement a sifting process on the instances to exclude all those with missing channels. After that, we convert the resulting cleaned dataset into strings for easily porting the timestamps into MATLAB. Once we terminate the import phase, MATLAB resamples the data at a frequency of 20 Hz, as declared for the WISDM dataset^25,26^. Then, we filter the signal by removing undesired frequency components. The results are then converted back to text for a final reprocessing and reorganization step using Python, which yields the released version of the dataset.

### Spike-encoded SIDDHA

In addition to the raw data, the SIDDHA dataset includes a spike-encoded version. Specifically, we propose an example of configuration for 11 distinct encoding techniques^30^: Poisson rate (PR), threshold-based representation (TBR), moving window (MW), step-forward (SF), zero-crossing step-forward (ZCSF), Hough spike algorithm (HSA), modified Hough spike algorithm (MHSA), Bens spiker algorithm (BSA), phase encoding (PE), time-to-first-spike (TTFS), and burst encoding (BE). The code to reproduce such encoding configurations and to explore possible alternative settings is available through the spikify library at https://spikify.readthedocs.io/en/latest/.

We reduced all reconstructed instances to 3500 timestamps to produce the spike-based data version for two main reasons. Firstly, with this simplification, we ensure a uniform length across all dataset instances and a consequent easier handling for spiking neural network (SNN) oriented users. Secondly, we can consistently apply the bin-based encoding techniques.

To produce the spike-encoded data available in the SIDDHA dataset, we selected some specific parameters. Table 2 illustrates their meaning and values.

**Table 2:** Summary of the encoding parameters for each algorithm used to produce the spike-encoded SIDDHA.

Encoding	Parameters
PR	*r*: 20
TBR	*γ*: 0.5
MW	*window size*: 3, *threshold*: mean of variation of the signal
SF, ZCSF	*jump*: maximum-to-minimum differences for each channel, *γ*: 10
HSA	*filter*: boxcar, *size*: 3
MHSA	*filter*: boxcar, *size*: 3, *threshold*: 0.85
BSA	*filter*: boxcar, *size*: 3,*threshold*: 1
PE	*β*: 5
TTFS	*bins*: 10
BE	*N*_*max*_: 5, *t*_*min*_: 0; *t*_*max*_: 4

## Data Records

The SIDDHA^31^ dataset is available through Hugging Face, with an open source license BSD-License (3-clause), at the following link: 10.57967/hf/7708. The data is stored in the archive folder in two formats: binary (extension .bin) and text (extension .txt).

The binary format, obtained through the Python data structures, includes the data from the smartphone and smartwatch device in a single file. We organize this information as a dictionary with access keys corresponding to the *phone* and *watch* data fields. For each entry, 51 dictionaries, namely one for each subject, are present, and the access key is the integer value between 0 and 50 that corresponds to the identification number of that subject. Compared to the WISDM dataset^25,26^, the prefix 16 is no longer present. The subsequent level includes the classes with keys corresponding to the labels used in the WISDM dataset^25,26^. The final level consists of the accelerometer and gyroscope measurements, accessible via the *acce* and *gyro* keys, respectively. Four lists organize data for both sensors as time, x-axis, y-axis, and z-axis values. The corresponding file is in the directory datasets/dataset/dataset.bin.

We arrange the text format into two folders, designated as datasets/dataset/dataset/phone and datasets/dataset/dataset/watch. Each folder contains 51 .txt files in which each line includes the following features separated by a semicolon: activity label, timestamp, x-, y-, and z-axes data from accelerometer, x-, y-, and z-axes data from gyroscope, for example we have the file data_phone_0.txt in the folder datasets/dataset/dataset/phone which contains the *phone* data collected by user id 0.

We locate the spike-encoded versions in the datasets/dataset_spike/dataset_spike folder. This folder contains the results from the 11 encoding techniques previously described in the file format .bin indicate by the wording spike_pr.bin, spike_tbr.bin, spike_mw.bin, spike_sf.bin, spike_zcsf.bin, spike_bsa.bin, spike_hsa.bin, spike_mhsa.bin, spike_pe.bin, spike_be.bin, spike_ttfs.bin.

As example the TBR encoded is in the file datasets/dataset_spike/spike_tbr.bin, and the format .txt in the file datasets/dataset_spike/spike_tbr/phone/data_phone_0.txt. The structure of the files is identical to that of the reconstructed dataset, with the only addition of the encoding technique to label the folders. The following are examples: spike_pr, in the case of Poisson rate encoding, and spike_tbr, for the threshold-based representation. Within the encoded data, Boolean variables, with values True and False, represent spikes.

**Fig. 2:**
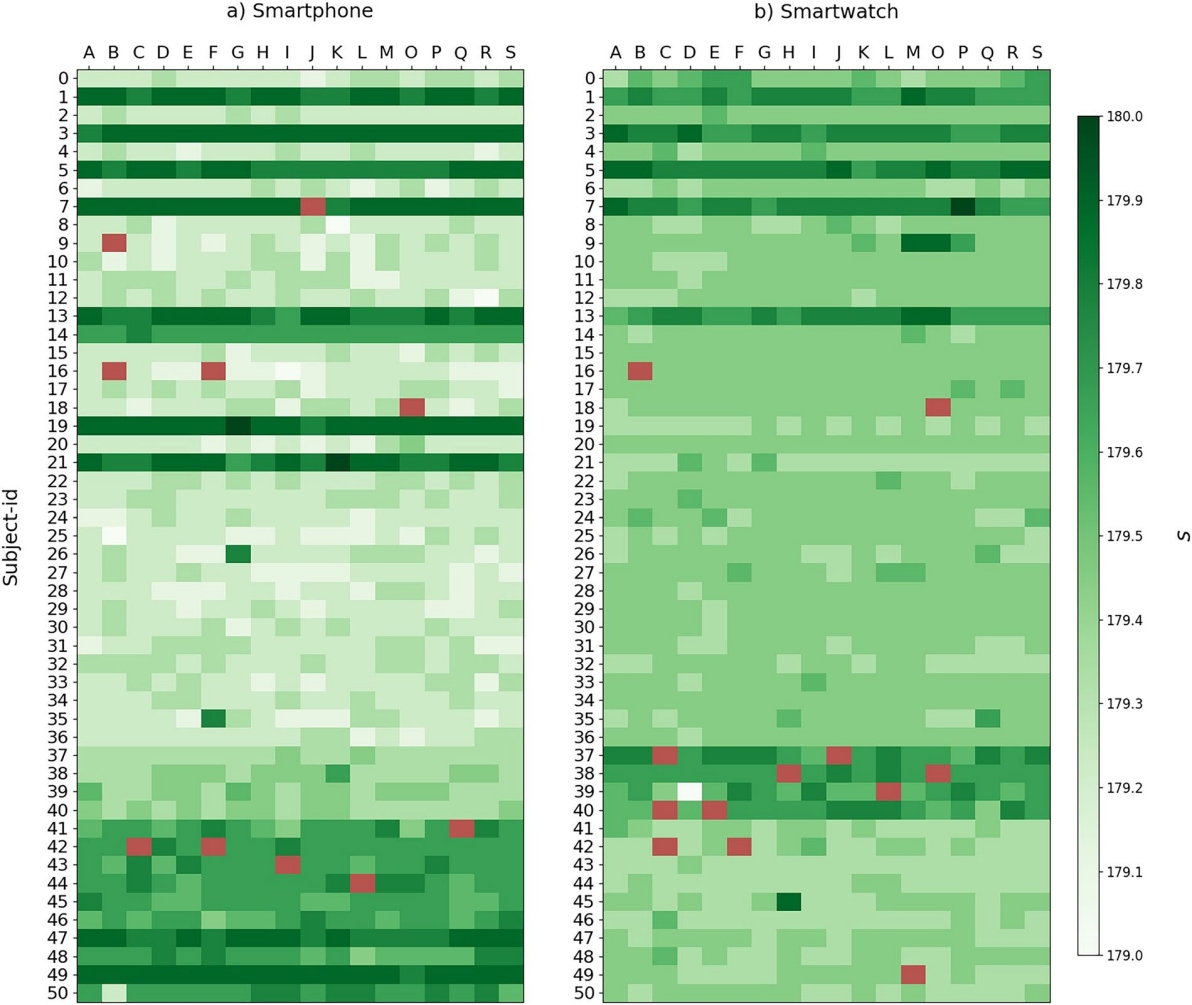
Overview of the sensor signals recorded via smartphone (**a**) and smartwatch (**b**) sorted by subject and class. The heat maps allow the identification of the duration of instances collected per subject identified by the ID and class, along with their label. The color scale represents the total length in seconds, as shown by the color bar to the side, while a different color indicates the absence of measurement.

Figure 2 illustrates the classes available for each subject, using a heatmap where the color intensity represents the time in seconds for each instance. As shown by the scale to the side, the measured time ranges from a minimum of 179.0 s to a maximum of 180.0 s. As previously mentioned, we discovered that specific subjects lack certain classes. We highlight this characteristic in Figure 2, adopting different colors. Specifically, focusing first on smartphone data, subject 7 lacks class J; 9 class B; 16 classes B and F; 18 class O; 41 class Q; 42 classes C and F; 43 class I; 44 class L. Concerning the smartwatch data, subject 16 lacks class B; 18 class O; 37 classes C and J; 38 classes H and O; 39 class L; 40 classes C and E; 42 classes C and F; and 49 class M.

## Technical Validation

We performed an in-depth validation of the SIDDHA dataset along three pathways: entropy, frequency distribution, and intensity distribution.

**Fig. 3:**
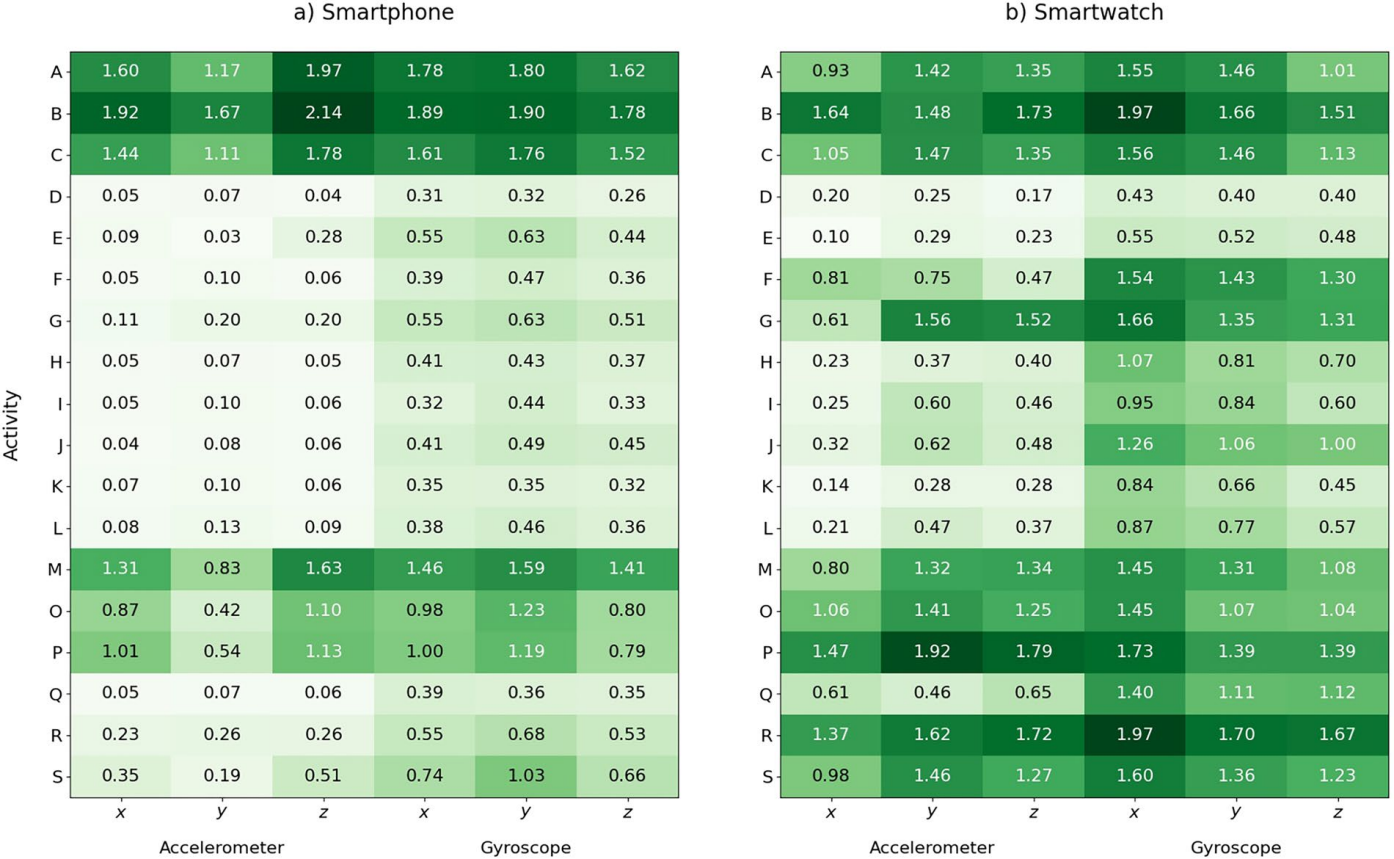
Heatmaps of entropy measurements for smartphone (**a**) and smartwatch (**b**). The heatmaps illustrate the entropy values of the sensor signals. These values show that the accelerometer signals have lower entropy than the gyroscope signals in both devices.

We quantify the information contained within the classes by utilizing the entropy measure following the Shannon definition^32^. Figure 3 provides a visual representation of it for both smartphone and smartwatch data, with a heatmap offering a summary of the values for all activities for each sensor channel or axis. A comparison of the entropy distributions for the two smart devices reveals different information content, with measurements obtained from the gyroscope sensor having a higher value for both smartphone and smartwatch data. However, we can observe some exceptions, with the A, B, C, M, O, and P classes having similar entropy values from the two sensors for both the smartphone and the smartwatch. These classes correspond to the non-hand-oriented activities “walking”, “jogging”, “stairs”, “kicking (soccer ball)”, and hand-oriented activities “playing catch with a tennis ball”, “dribbling (basketball)”.

**Fig. 4:**
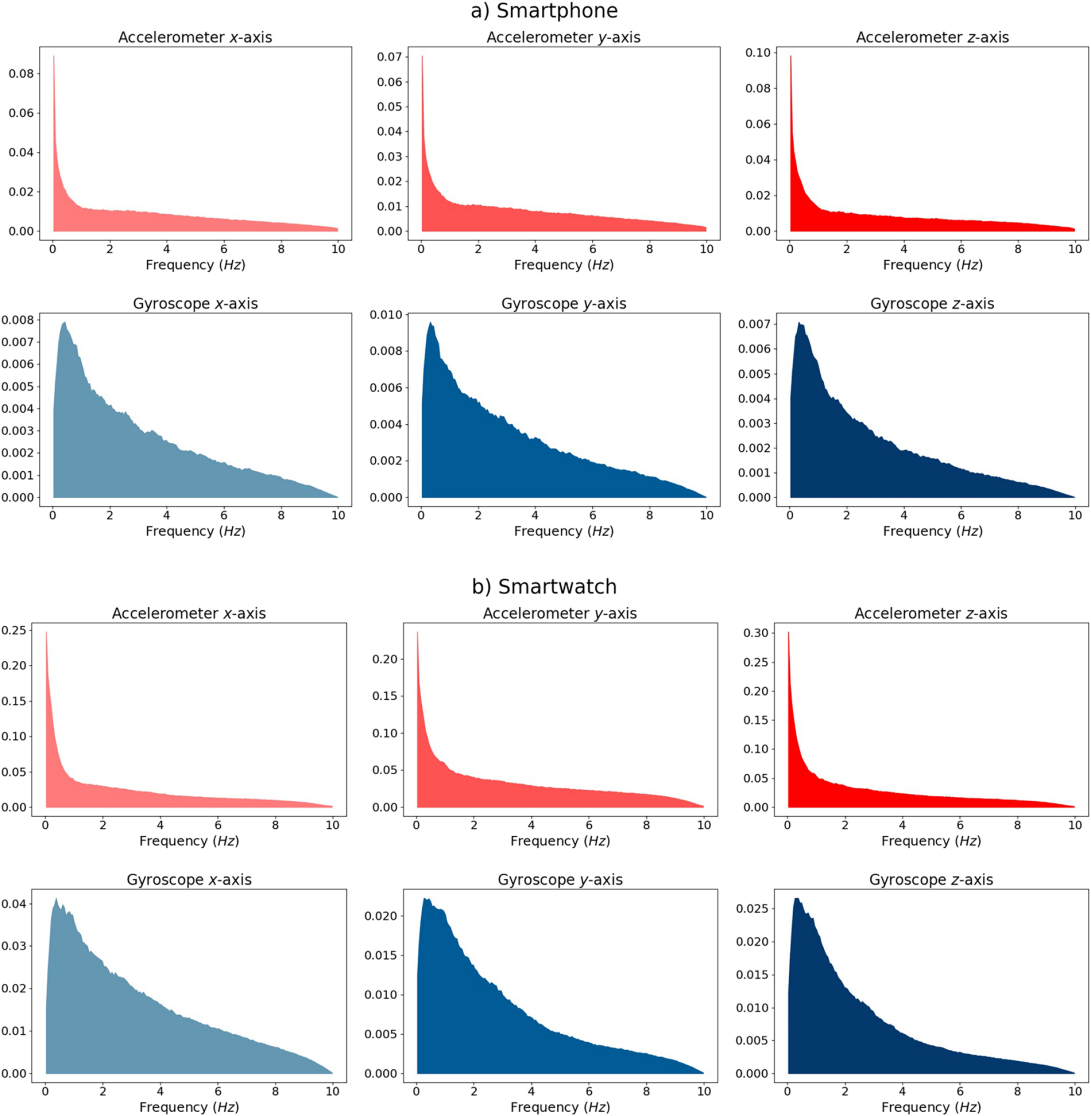
The plots illustrate a comparison of the frequency spectrum for smartphone (**a**) and smartwatch (**b**). The spectra are obtained through the median of the Fourier transform on all the available classes of users that compose the dataset. The accelerometer and gyroscope are reported at the top and bottom of Figures (**a**) and (**b**), respectively. In this case, a wider distribution is observed for the spectrum belonging to the gyroscope sensor.

The higher entropy level within gyroscope signals is inherently connected to the frequency spectrum in a mutual cause-and-effect relationship. As can be observed in Figure 4a and b for smartphone and smartwatch data, respectively, the intensity of the frequency components of signals originating from the gyroscope is more spread in the frequency domain up to the stop band at 10 Hz. On the contrary, the analysis of the accelerometer signals reveals that their spectrum has a remarkably sharper distribution at lower frequencies.

**Fig. 5:**
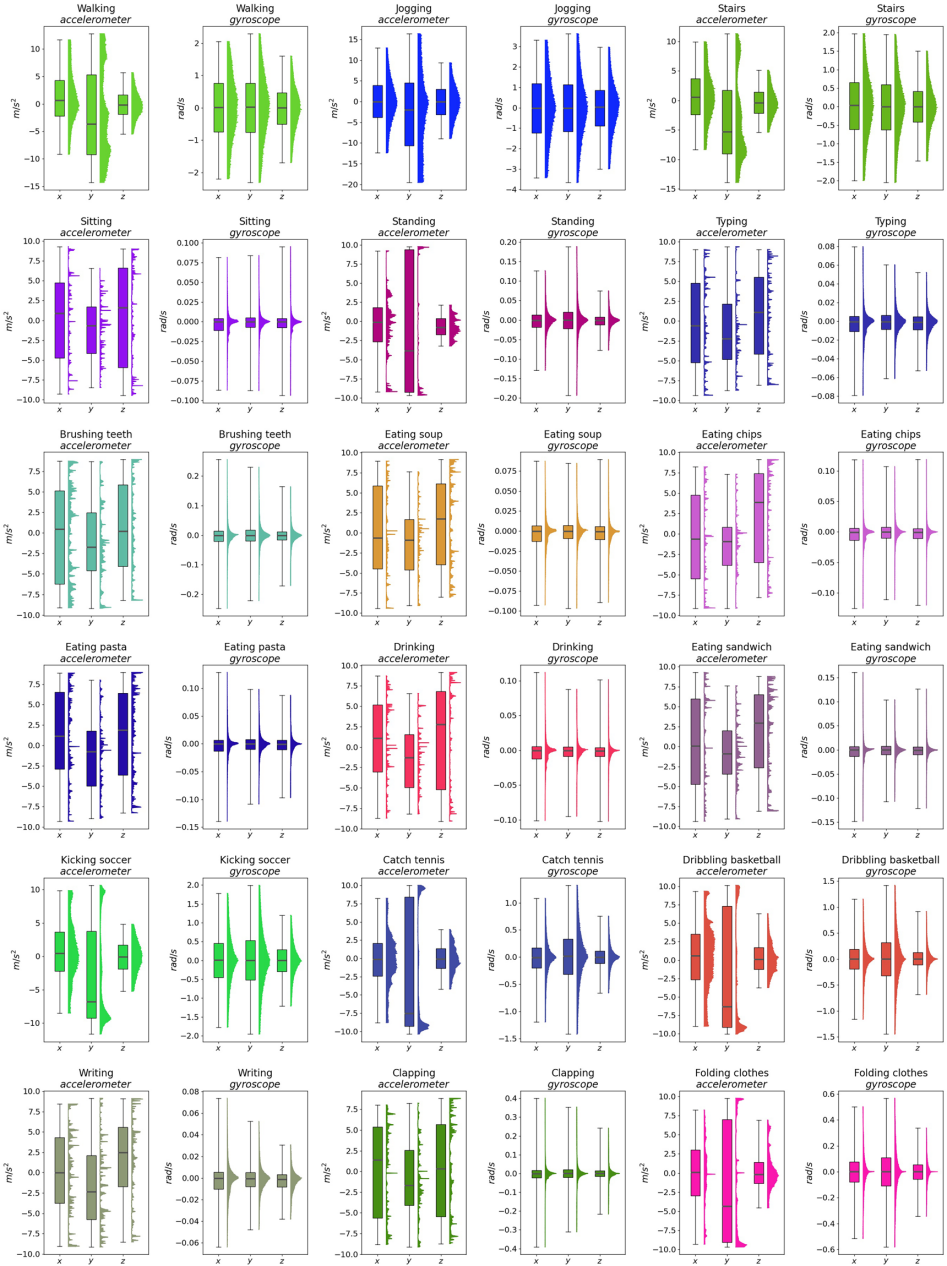
The plots show the intensity distribution of the accelerometer and gyroscope measurements for each class recorded with a smartphone. We derived these values by analyzing the signals of each class across different subjects. Each class contains paired accelerometer and gyroscope graphics, with the three measurements plotted along the x-, y-, and z-axes.

**Fig. 6:**
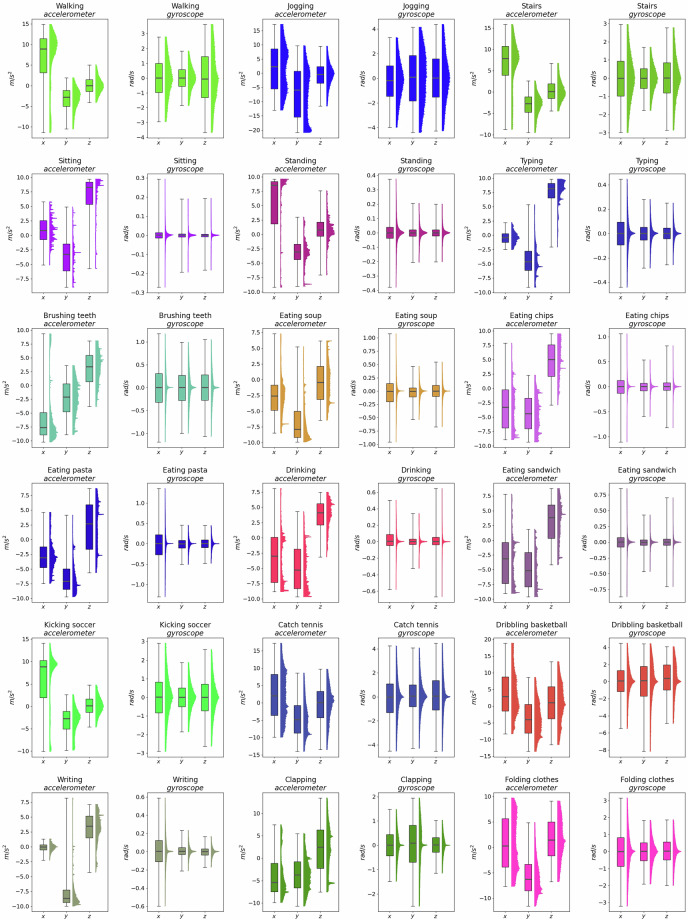
The graphics illustrate the intensity distribution of the accelerometer and gyroscope measurements for each class recorded with a smartwatch. We obtained these values by analyzing the signals for each class across multiple subjects. Each class has a set of accelerometer and gyroscope graphics showing measurements along the x-, y-, and z-axes.

Figures 5 and 6 show the distribution intensity for the 18 activities, including the ones recorded with the smartphone and the smartwatch, respectively. Each pair of graphics with the same color shows the signal intensity distribution for the accelerometer and gyroscope sensor, respectively. We adopted a boxplot representation, coupled with the corresponding distribution profile. A brief analysis suggests that each class exhibits a distinct scale and intensity distribution, underscoring the richness of the data present within the various instances. Focusing on the signals from the accelerometer, they present a broader intensity distribution than those acquired by the gyroscope, except for non-hand-oriented activities. At the device level, smartwatch data generally has a larger range of values than smartphone data, for both the accelerometer and the gyroscope readings.

In order to facilitate the comparison of the following analysis with the measurements from the SIDDHA and WISDM datasets, the same metrics were employed to characterise the WISDM samples, which are available in the Supplementary Information.

### Usage Example

As an example to validate the effectiveness of the reconstruction strategy designed and adopted to produce the SIDDHA dataset, we addressed the classification task in the forms proposed by Gupta^33^ and by Fra *et al*.^34^, replicating the experimental procedures for both the original WISDM data^25,26^ and the SIDDHA data. In addition to direct comparison with previous works specifically targeting HAR on the WISDM dataset^25,26^, we also trained the Legendre memory unit (LMU) architecture directly from its original paper^35^.

As it is reported in Table 3, the SIDDHA dataset allows for gains in classification accuracy for both the whole dataset and the general hand-oriented subset of activities. Although the LMU demonstrates slightly lower performance on the full SIDDHA dataset, it has a performance gain on the considered subset.

**Table 3:** Comparison of classification results between the WISDM dataset^25,26^ and our SIDDHA dataset. Recurrent architectures only have been considered given the temporal character of the data.

Ref.	Model	Dataset	Subset	Accuracy
^33^	GRU	*WISDM*	Whole dataset	91.42%
		SIDDHA		91.93%
^33^	LSTM	*WISDM*	Whole dataset	90.22%
		SIDDHA		91.78%
^35^	LMU	*WISDM*	Whole dataset	83.59%
		SIDDHA		82.15%
^34^	LSTM	*WISDM*	General, hand-oriented	95.61%
		SIDDHA		96.14%
^34^	LMU	*WISDM*	General, hand-oriented	93.03%
		SIDDHA		93.65%

## Usage Notes

This work presents the Smart Inertial Device Data from Human Activities (SIDDHA) dataset, a reconstructed version of the Smartphone and Smartwatch Activity and Biometrics Dataset by the WISDM Lab^25,26^. SIDDHA contains signals from imu embedded in smartwatch and smartphone, and it can be used as training data for classification applications of time-dependent signals for body monitoring in the field of ML and artificial intelligence of things (AIoT).

## Supplementary information

Supplementary Information

## Data Availability

The SIDDHA^31^ dataset is publicly available on Hugging Face at 10.57967/hf/7708.

## Code availability

The code for reconstructing the instances can be found in a dedicated Hugging Face repository at 10.57967/hf/7708, specifically in the resample_script folder. The Python scripts included are the following: resample_script/1-preprocessing.py, to simplify the analysis of the original dataset and the creation of its binary version; resample_script/2-anomaly_check.py, to eliminate instances with partial measurements from the original dataset; resample_script/3-matlab_unpack.py, to create a textual version of the data that can be processed by the MATLAB resample_script/reconstruction.m script (whose main task is to reconstruct the data and apply the filtering operation); resample_script/4-matlab_repack.py, to reorganize the reconstructed data in binary and textual formats.

In order to develop the reconstruction process of the SIDDHA dataset, the execution of the process was developed using Python 3.12.4, Numpy 2.0.2 and Matplotlib 3.10.0. The version of Matlab used is R2025b. All the Python dependencies are listed in the environment.yml file available in the repository.
