# Supplementary material for: The Smart Inertial Device Data from Human Activities dataset
Source: Sci Data. 2026 Apr 9;13:864. doi: 10.1038/s41597-026-06860-w (PMC13250143; doi:10.1038/s41597-026-06860-w)
Supplement: Supplementary file 1 — Supplementary Information [file 41597_2026_6860_MOESM1_ESM.pdf]

Supplementary Information

The Smart Inertial Device Data from Human Activities dataset

Riccardo Pignari[1,*], Bendetto Leto[1], Stefano Quer[1], Enrico Macii[1], Gianvito Urgese[1], and Vittorio Fra[1]

[1]Politecnico di Torino, Turin, 10129, Italy
[*]corresponding author: Riccardo Pignari (riccardo.pignari@polito.it)

Additional characterization of original data

This supplementary section provides an expanded set of evaluation metrics computed on the original WISDM dataset, with the aim of offering a more detailed characterization of the underlying sensor signals. In addition to the analyses reported in the main paper, here we include complementary metrics that help elucidate the temporal, spectral, and statistical properties of the data.

First, we report entropy-based measures that capture the intrinsic variability and complexity of the accelerometer and gyroscope signals across different classes and devices. These metrics allow for an assessment of the information content present in the raw sensor streams. We then examine the frequency-domain behavior of the dataset through the analysis of the Fourier-based spectra of each sensor channel. This provides insights into the dominant frequency components associated with different activities and highlights differences between devices and sensing modalities. Finally, we include intensity-based measurements derived from the raw tri-axial signals, offering a direct view of signal amplitude distributions across subjects and classes.

Together, these complementary evaluations offer an overview of the original dataset characteristics, supporting reproducibility and facilitating deeper interpretation of the experimental results presented in the main paper.

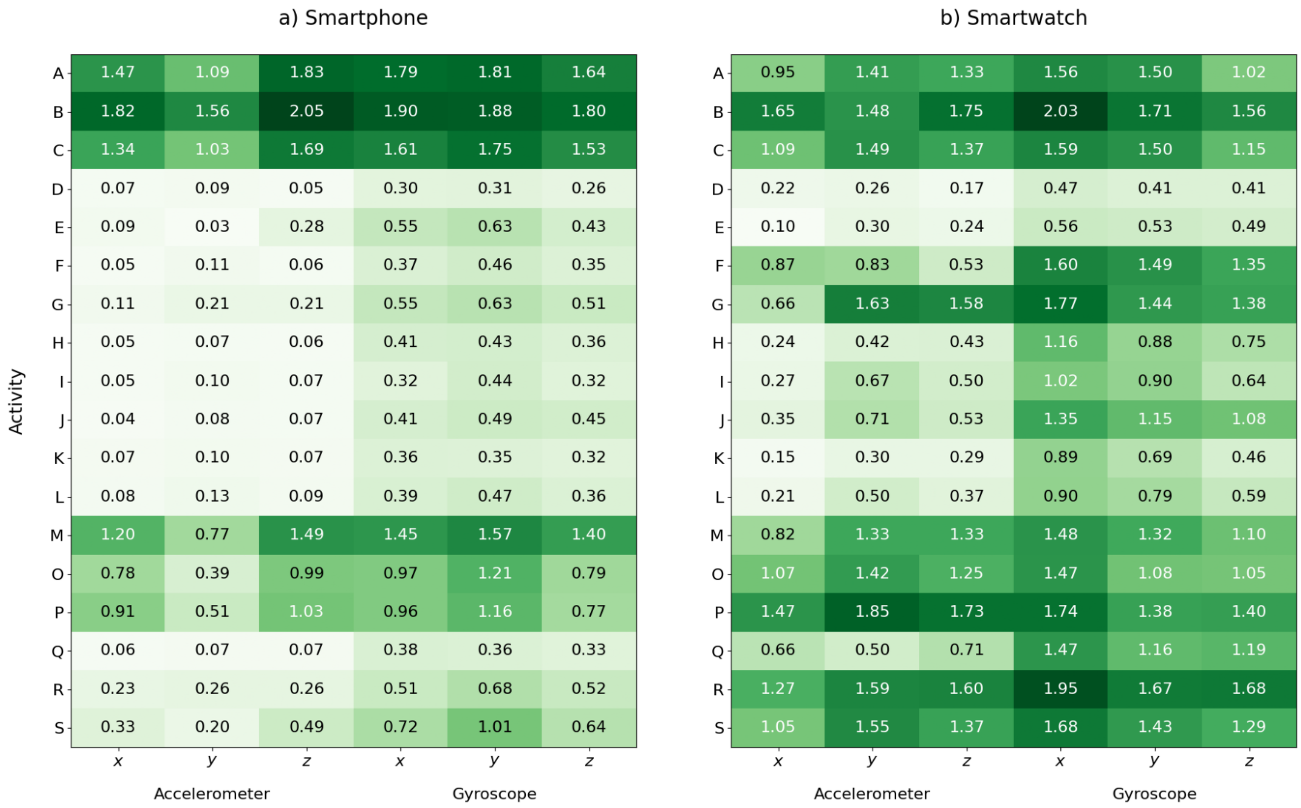

Figure 1. Heatmaps of entropy measurements for the smartphone (*a*) and smartwatch (*b*). The maps display the entropy values computed from the sensor signals, showing that accelerometer readings exhibit consistently lower entropy than gyroscope readings for both devices.

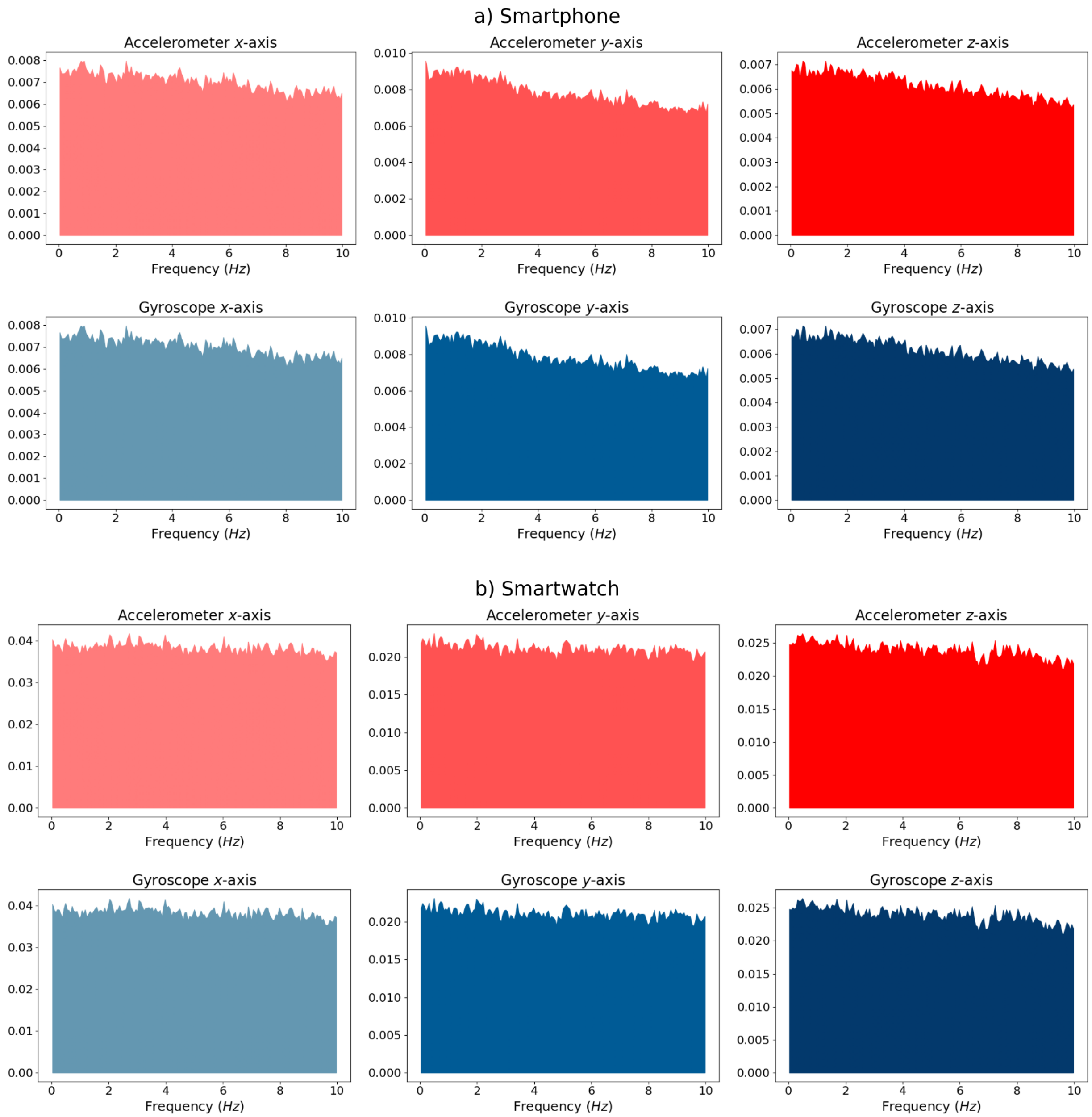

Figure 2. Frequency-spectrum comparison for the smartphone (*a*) and smartwatch (*b*). Each spectrum is obtained by computing the median of the Non-Uniform Fast Fourier Transform across all user classes in the dataset. For both devices, accelerometer signals are shown in the upper panels and gyroscope signals in the lower panels.

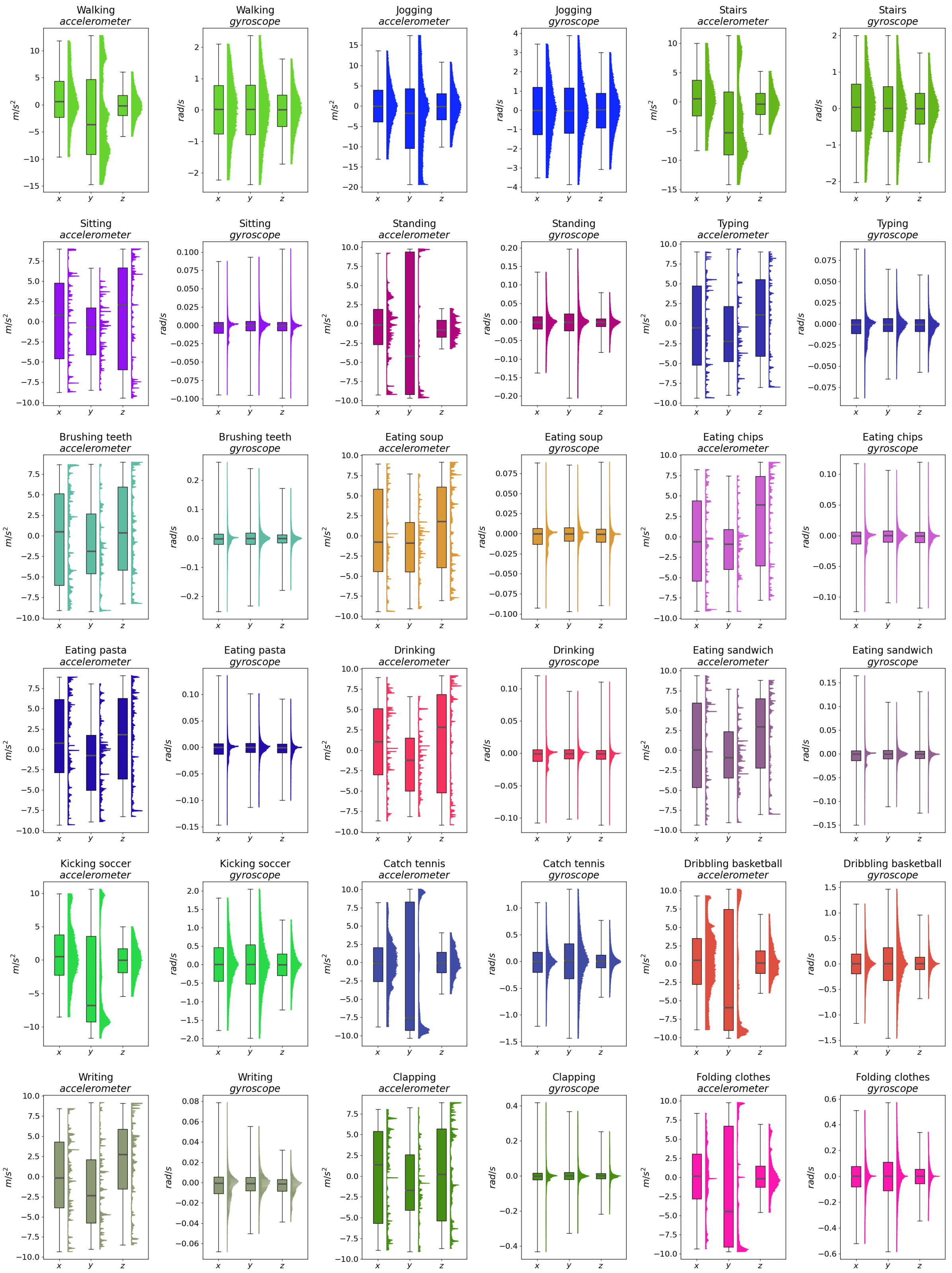

Figure 3. Intensity distributions of accelerometer and gyroscope measurements for each class recorded with the smartphone. The values are obtained by analyzing signals from all subjects within each class. For every class, paired accelerometer and gyroscope plots are provided, with the three signal components displayed along the x-, y-, and z-axes.

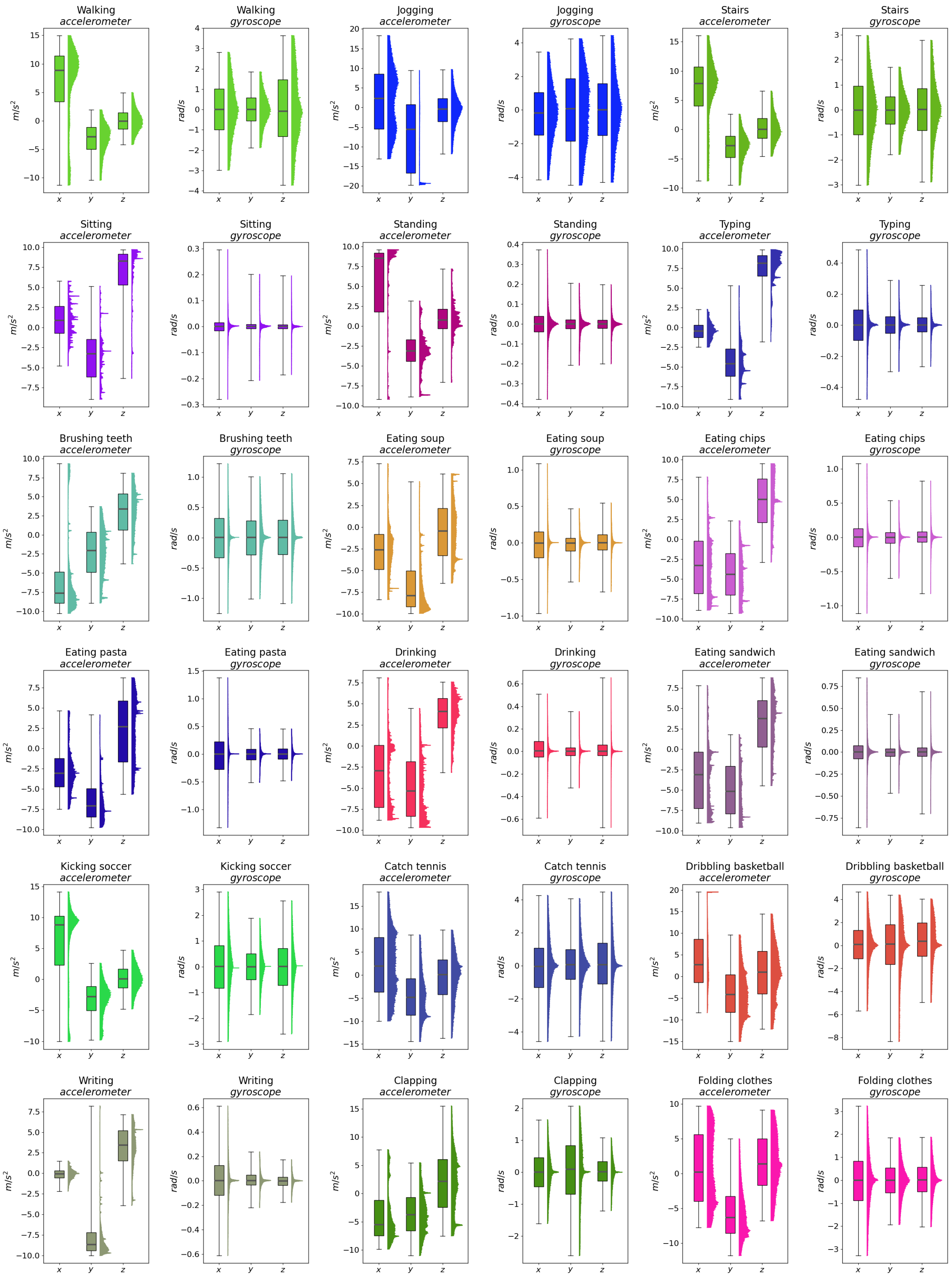

Figure 4. Intensity distributions of accelerometer and gyroscope measurements for each class recorded with the smartwatch. The values are derived by analyzing the signals from multiple subjects within each class. For each class, paired accelerometer and gyroscope plots are shown, with measurements reported along the x-, y-, and z-axes.
